# Heterozygous Mutation of *Drosophila Opa1* Causes the Development of Multiple Organ Abnormalities in an Age-Dependent and Organ-Specific Manner

**DOI:** 10.1371/journal.pone.0006867

**Published:** 2009-08-31

**Authors:** Parvin Shahrestani, Hung-Tat Leung, Phung Khanh Le, William L. Pak, Stephanie Tse, Karen Ocorr, Taosheng Huang

**Affiliations:** 1 Ecology and Evolution, University of California Irvine, Irvine, California, United States of America; 2 Department of Biological Sciences, Purdue University, West Lafayette, Indiana, United States of America; 3 Division of Human Genetics, Department of Pediatrics, University of California Irvine, Irvine, California, United States of America; 4 Burnham Institute for Medical Research, Program for Systems and Developmental Biology, Center for Neuroscienes and Aging, La Jolla, California, United States of America; 5 Department of Developmental and Cell Biology, University of California Irvine, Irvine, California, United States of America; 6 Department of Pathology, University of California Irvine, Irvine, California, United States of America; Stanford University School of Medicine, United States of America

## Abstract

Optic Atrophy 1 (OPA1) is a ubiquitously expressed dynamin-like GTPase in the inner mitochondrial membrane. It plays important roles in mitochondrial fusion, apoptosis, reactive oxygen species (ROS) and ATP production. Mutations of *OPA1* result in autosomal dominant optic atrophy (DOA). The molecular mechanisms by which link OPA1 mutations and DOA are not fully understood. Recently, we created a *Drosophila* model to study the pathogenesis of optic atrophy. Heterozygous mutation of *Drosophila OPA1* (*dOpa1*) by P-element insertion results in no obvious morphological abnormalities, whereas homozygous mutation is embryonic lethal. In eye-specific somatic clones, homozygous mutation of *dOpa1* causes rough (mispatterning) and glossy (decreased lens deposition) eye phenotypes in adult *Drosophila*. In humans, heterozygous mutations in *OPA1* have been associated with mitochondrial dysfunction, which is predicted to affect multiple organs. In this study, we demonstrated that heterozygous *dOpa1* mutation perturbs the visual function and an ERG profile of the *Drosophila* compound eye. We independently showed that antioxidants delayed the onset of mutant phenotypes in ERG and improved larval vision function in phototaxis assay. Furthermore, heterozygous *dOpa1* mutation also caused decreased heart rate, increased heart arrhythmia, and poor tolerance to stress induced by electrical pacing. However, antioxidants had no effects on the dysfunctional heart of heterozygous *dOpa1* mutants. Under stress, heterozygous *dOpa1* mutations caused reduced escape response, suggesting abnormal function of the skeletal muscles. Our results suggest that heterozygous mutation of *dOpa1* shows organ-specific pathogenesis and is associated with multiple organ abnormalities in an age-dependent and organ-specific manner.

## Introduction

OPA1 is encoded by a ubiquitously expressed nuclear gene and localized in the inner mitochondrial membrane [1]–[3]. OPA1 consists of a mitochondrial targeting signal (MTS), a transmembrane domain (TMD), a presenilin-associated rhomboid-like protease (PARL) site, and a dynamin/GTPase domain. The PARL can process OPA1, converting the insoluble inner mitochondrial membrane protein to a soluble protein [1], [3]. OPA1 also regulates cytochrome-c mediated apoptosis by modulating mitochondrial cristae morphology [4], [5], [6], [7]. In humans, mutation of OPA1 causes retinal ganglion cell death and optic atrophy [4]. The disease leads to loss of central vision and color vision abnormalities [5]–[8]. More than 100 different OPA1 mutations have been identified, including missense, nonsense, deletion/insertion and splicing mutations [1], [2], [13], [14]. A few families with sex-influenced DOA phenotypes have been described [9]. In a study of the sex-influenced phenotype, we found that vision loss was more severe among affected males than females, and caused by the mutation IVS9 +2A>G in the *OPA1* gene [16], [17].

Previous studies suggest that OPA1 mutations may cause multiple organ anomalies. This is supported by several lines of evidence. First, OPA1 is a ubiquitously expressed protein [1], [3]. When mutated, it results in mitochondrial fragmentation, a decreased mitochondrial membrane potential and decreased ATP production. The impaired ATP synthesis is associated with decreased oxygen consumption [10]. Second, OPA1 mutations affect organs other than the retina [18], [19], [20], [21], [22], [23], [24], [25], [26]. For example, the R445H mutation causes both optic atrophy and hearing loss [11]–[16]. The G401D missense mutation was identified in a family with optic atrophy and hearing loss, suggesting that optic atrophy and hearing loss are not R445H mutation-specific clinical phenotypes. Indeed, it has been reported that OPA1 mutations are also associated with ptosis and ophthalmoplegia [27], [28], [29]. Third, in other mitochondrial diseases, such as myoclonus epilepsy associated with ragged-red fibers (MERRF), mitochondrial myopathy, encephalopathy, lactic acidosis and stroke-like episodes (MELAS), mitochondrion are fragmented which is associated with proteolytical processing of OPA1[17]. All of these suggest that *OPA1* mutation could cause multiple organ abnormalities [17] and that analysis of the major organ systems may extend our knowledge of the clinical manifestations of *OPA1* mutations.

We have developed a *Drosophila* model of optic atrophy [18]. There are several advantages to using *Drosophila* models for studying eye disorders: 1) significant cost and time savings; 2) eye phenotypes are easier to detect in *Drosophila* than in other models; 3) the *Drosophila* eye is non-essential for viability. Versatile technologies exist for generating, identifying and characterizing mutations in the *Drosophila* retina, making this an unrivaled system for deciphering gene function. In addition, there is a high degree of similarity between *Drosophila* Opa1 and human OPA1[18]. Recently, we generated a *Drosophila* knockout model for optic atrophy. Heterozygous mutation of *dOpa1* induced by a P-element or transposon insertion caused no structural abnormalities under a light microscope, whereas homozygous mutation resulted in embryonic lethality. In the eye-specific somatic clones, homozygous mutation of *dOpa1* caused rough (mispatterning) and glossy (decreased lens deposition) eye phenotypes in adult *Drosophila* and is associated with increased ROS production [18]. Since OPA1 mutations in humans cause autosomal dominant phenotypes and heterozygote mutations display phenotypes, the grossly normal eye in heterozygous mutant *Drosophila* under a light microscope could still have abnormal function and warrant further analysis.

To study OPA1 function and whether the loss of function is linked to the development of multiple organ abnormalities, we determined if heterozygous *dOpa1* mutation affects the function of multiple organs and the underlying mechanisms in *Drosophila*. Specifically, we examined eye, heart and skeletal muscle in heterozygous *dOpa1 Drosophila* mutants. Our result showed that heterozygous *dOpa1* mutation resulted in abnormal electroretinograms (ERG) in an age-dependent manner. Abnormal visual function was also demonstrated in a phototxis assay. The abnormal ERG and visual dysfunction could be partially rescued by antioxidant treatment. Heterozygous *dOpa1 Drosophila* mutants also showed reduced heart rate, increased heart arrhythmias, and poor heart function as indicated by decreases in fractional shortening and increased heart failure in response to electrical pacing. Additionally, under heat shock stress, the heterozygous mutants showed reduced escape response suggesting reduced muscle function. Our results suggest that heterozygous mutation of *dOpa1* could cause multiple organ abnormalities and that ROS may play a role in the development of some organs, but not others, suggesting that the pathogenesis could be organ specific.

## Results

### Heterozygous *dOpa1* mutation results in loss of normal eye function in adult *Drosophila* and antioxidants can partially prevent the loss of function

**Figure 1:**
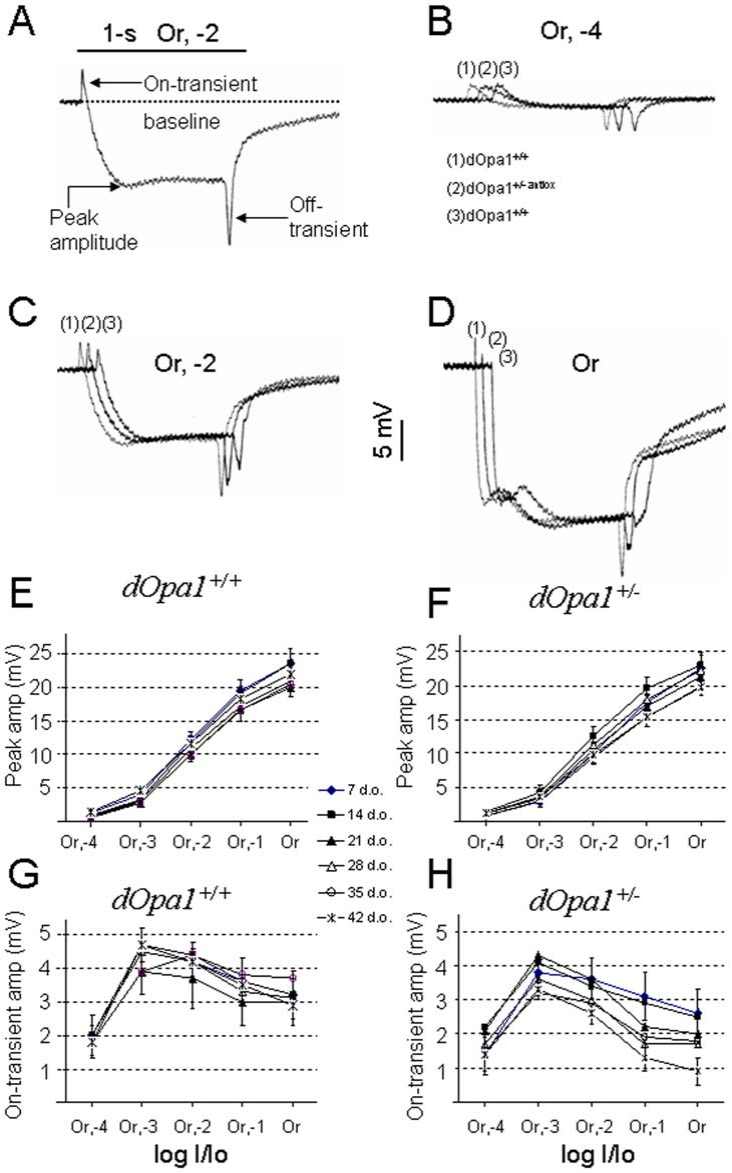
Electroretinogram analyses show an age-dependent reduction in the on-transient but not in the phototransduction response in heterozygous *dOpa1 (dOpa1^+/−^)* mutants. (A) A typical ERG response showing on-/off-transients and peak amplitude when elicited with an Or, -2 stimulus. (B) Typical ERGs obtained from 42 d.o. *dOpa1^+/+^*, *dOpa1^+/−antiox^* and *dOpa1^+/−^* using Or, -4 light stimuli are superimposed. The on-transients and peak amplitudes from the three *Drosophila* types are comparable. (C) Typical ERGs from 42 d.o. *dOpa1^+/+^*, *dOpa1^+/−antiox^* and *dOpa1^+/−^* during light stimulus with Or, -2 were superimposed. The on-transient, but not the peak amplitudes, from *dOpa1^+/−^* were smaller than those of *dOpa1^+/+^* and *dOpa1^+/−antiox^*. (D) Typical ERGs from 42 d.o. *dOpa1^+/+^*, *dOpa1^+/−antiox^* and *dOpa1^+/−^* during light stimulus with Or were superimposed. The on-transients of *dOpa1^+/−^* and *dOpa1^+/−antiox^*, but not the peak amplitudes, were significantly smaller than those of *dOpa1^+/+^*. (E) V-log I curves and (G) on-transient amplitudes vs log I from 7, 14, 21, 28, 35 and 42 d.o. *dOpa1^+/+^* show no age-dependent reduction in on-transient and peak amplitude responses of the photoreceptor component. V-log I curves from 7, 14, 21,28, 35, and 42 d.o. *dOpa1^+/−^* show no age-dependent reduction in peak amplitude responses (F) while the on-transient amplitudes vs log I curves from *dOpa1^+/−^* show an age-dependent reduction in on-transient amplitudes (H).

Previously, we have analyzed the effects of *dOpa1* mutation in *Drosophila* eyes. Loss of a single copy of *dOpa1* did not elicit a gross eye phenotype other than morphologically perturbed mitochondria in transmission electron microscopy (TEM) [18]. To identify subtle phenotypes, a large cohort of *dOpa1^+/−^* and *dOpa1^+/+^* control *Drosophila* were aged and their ERG profiles measured every 7 days for 6 weeks. As shown in Figure 1, heterozygous mutation of *dOpa1* resulted in perturbed ERG profiles in an age-dependent manner. The *dOpa1^+/−^* mutants showed an age-dependent progressively worsening reduction in the on-/off-transients beginning at 28 d.o., but no reduction in the peak amplitude for the six weeks tested (Figure 1). Since the on-transient, but not the off-transient, is independent of the stimulus duration, we decided to determine if there were defects in the age-dependence of the on-transient amplitudes in *dOpa1^+/+^* and *dOpa1^+/−^ Drosophila*. The ERG response in *dOpa1^+/+^* flies at 42 d.o., typically showed an initial transient response (the on-transient) and then a sustained corneal-negative photoreceptor response followed by an off-transient when the light stimulus was turned off. ERGs with different intensities of orange light stimuli, Or,-4 (B), Or,-2 (C), and Or (D), are superimposed to show the differences in the on-transient amplitudes. There was a severe reduction in the on-transient amplitude in *dOpa1^+/−^* eyes with brighter Or (D), but not with a dimmer stimulus (B). Our data showed that in *dOpa1^+/+^*, there was no significant age-dependent reduction in peak (E) or on-transient amplitudes (G) over the range of light stimuli tested. However, in *dOpa1^+/−^*, there was a significant age-dependent reduction in on-transient amplitudes (H) but not in peak amplitudes (F). Since the on-transient has been shown to be originated from the laminar monopolar L1 and L2 neurons [19], [20], these results suggest that the *dOpa1^+/−^* mutation has no apparent effect on photoreceptor function but has an effect on laminar neuron function or synaptic transmission between the photoreceptor cells and their target laminar neurons.

**Figure 2:**
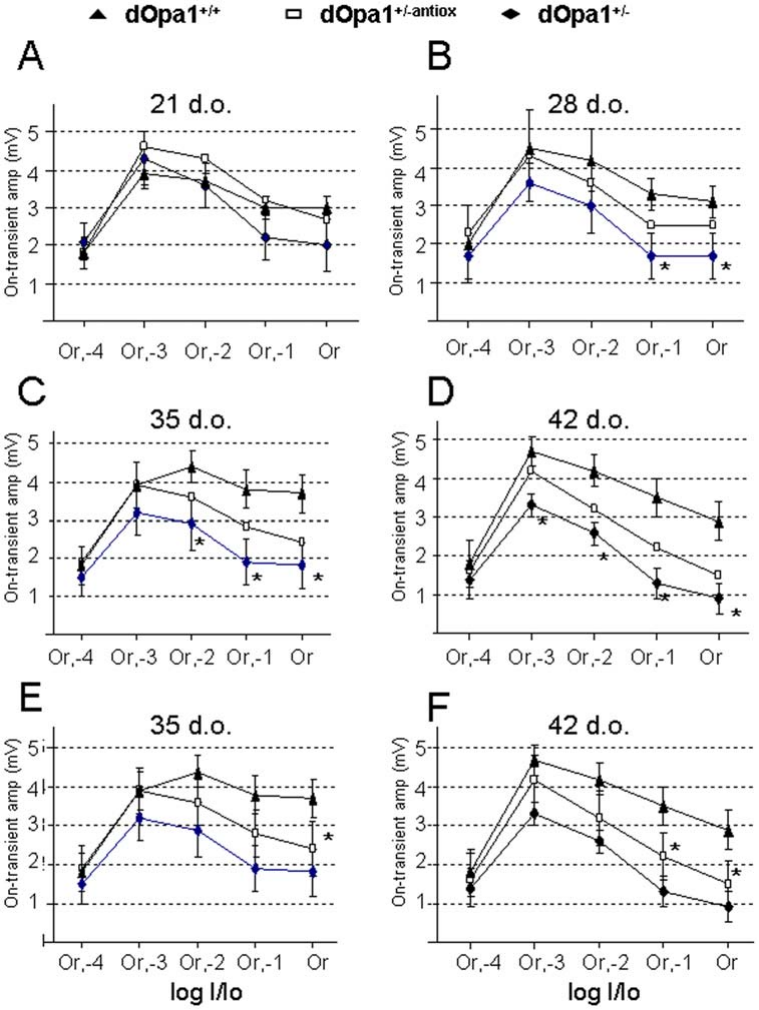
Heterozygous *dOpa1* mutation causes a progressive reduction in on-transient amplitudes in an age-dependent manner and antioxidants can reduce the progression. (A) *dOpa1^+/−^* shows no significant reduction in on-transient amplitudes when compared to *dOpa1^+/+^* at 7 to 21 d.o. (shown are the 21 d.o. data). *dOpa1^+/−^* shows significant reductions in on-transient amplitudes with the Or,-1 and Or stimuli at 28 d.o., (B) and with Or,-2, Or,-1, and Or stimuli at 35 d.o. (C), and at all Or light stimuli except the very dim Or,-4 at 42 d.o. (D) Antioxidant fed *dOpa1^+/−^* shows an increase in the mean on-transient amplitudes (A–D), but the increase is not significant when compared to *dOpa1^+/−^* (E & F, with error bars shown.). When compared to *dOpa1^+/+^*, at 35 d.o. antioxidant fed *dOpa1^+/−^* shows a significant reduction in on-transient amplitudes only with the Or stimulus (E), but not with Or,-2, and Or,-1 as in *dOpa1^+/−^* (C). At 42 d.o. *dOpa1^+/−antiox^* shows a significant reduction in the on-transient amplitudes only with Or,-1 and Or (F), but not with Or,-3, and Or,-2 as in *dOpa1^+/−^* (D). Or = Or light stimulus; Or, -1 = Or light stimulus with a log I/Io = -1 filter to reduced the orange light intensity by 10 fold; Or, -2 = Or light stimulus with intensity reduced by 100 fold, Or, -3 = 1,000 fold reduced Or light stimulus, and Or, -4 = 10,000 fold reduced Or light stimulus.

To study the effect of age in detail, on-transient amplitudes were compared among *dOpa1^+/−^ Drosophila* of different ages. There was an age-dependent progressive reduction of the on-transient amplitudes and the defect could be detected over a wider range of light stimuli in the older *dOpa1^+/−^ Drosophila* (Figure 2). At 28 d.o., the on-transient defect in *dOpa1^+/−^* was only seen with the two brightest light stimuli (B). At 35 d.o., *dOpa1^+/−^* showed significantly reduced on-transient amplitudes with Or,-2, Or,-1, and Or light stimuli (C). At 42 d.o., reduction in on-transient amplitudes was manifested in all but the dimmest stimulus in *dOpa1^+/−^* (D).

To test if the defect found could be reversed by antioxidant treatment, on-transient amplitudes were determined in 7 to 42 d.o. *Drosophila* that were fed with antioxidant-containing diet. As shown in Figure 2, antioxidant feeding ameliorated the age-dependent reduction in on-transient amplitudes in the *dOpa1^+/−^* mutant at all tested ages. Although the differences in on-transient amplitudes between *dOpa1^+/−^* and antioxidant fed *dOpa1^+/−^* (*dOpa1^+/−antiox^*) were non-significant, the latter consistently showed larger mean on-transient amplitudes at all light intensities tested and a slower reduction in on-transient amplitude with age when compared with *dOpa1^+/+^*. At 35 d.o., a significant reduction in on-transient amplitude was seen only with the brightest light stimulus in *dOpa1^+/−antiox^* (Figure 2E). At 42 d.o., *dOpa1^+/−antiox^* showed significantly reduced on-transient amplitudes at the brightest two Or stimuli (F), while *dOpa1^+/−^* showed a significant reduction over a wider range of Or light stimuli (D). Thus, antioxidant fed *dOpa1^+/−^* delayed the onset of on-transient defect by one to two weeks. These results suggest that ROS play a very important role in the pathogenesis of optic atrophy and that antioxidants are a potential therapeutic agent for this condition.

### Heterozygous *dOpa1* mutation causes visual abnormalities in phototaxis and antioxidants can partially prevent the visual loss

**Figure 3:**
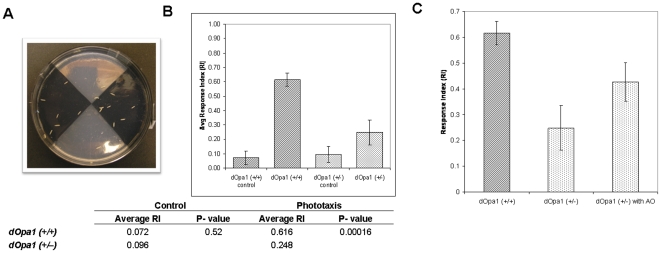
A. *Larval phototaxis assay*. A plastic petri plate was divided into four quadrants, two dark quadrants diametrically opposite to two clear quadrants. 1% agarose was used for the clear and 1% agarose containing 0.6% charcoal powder was used for the dark quadrants. 50 second instar larvae were placed at the center of the plate and were allowed to migrate for 3 minutes in complete darkness for the control, and in the presence of light by using a light box. The assay was repeated 3 times. Figure 3B. *Larval Phototaxis Response Index*. 50 second instars were used for each trial (total of 3 trials each). Wildtype *dOpa1^+/+^* exhibited strong negative phototatic response in the presence of light (RI = 0.616). Heterozygous mutant *dOpa1^+/in3^* showed little light response (RI = 0.248). Antioxidant treatment made no difference in wildtype *dOpa1^+/+^* (RI = 0.667). Mutant *dOpa1^+/in3^* treated with antioxidant exhibited a significantly stronger negative phototatic response to light (RI = 0.427).

To test if heterozygous *dOpa1* mutation affects visual functions at an earlier stage of fly development, we examined the larval visual system in *dOpa1^+/−^* heterozygous mutants with a phototaxis assay. This assay allowed us to measure larval visual phototactic response to light (Figure 3A). Fifty second-instar larvae were tested in three trials. In the absence of light, average response indexes (RI) for *dOpa1^+/+^* and *dOpa1^+/−^* were similar (RI = 0.072, RI = 0.096, respectively, p>0.05) (Figure 3B). However, with light, *dOpa1^+/−^* mutants scattered on the plate showing little visual phototatic response (RI = 0.248), while wild-type *dOpa1^+/+^* exhibited strong negative phototactic behavior (RI = 0.616, p<0.05) (Figure 3B). These results were replicated using both *dOpa1^+/ex2^* and *dOpa1^+/in3^ Drosophila* as discussed in our earlier paper [18]. Our results suggest that heterozygous mutation of *dOpa1* causes loss of visual acuity in *Drosophila*. This observation is consistent with the results of ERG, which indicates heterozygous mutation of *dOpa1* affects laminar neuron function or synaptic transmission between the photoreceptor cells and their target laminar neurons.

To test if treatment with antioxidants can improve the visual function of *dOpa1^+/−^*heterozygous mutants, *dOpa1^+/+^* and *dOpa1^+/−^ Drosophila* were treated with antioxidant. Again, fifty second-instar larvae were collected and the phototaxis assay was repeated three times. In the absence of the light, average RI for *dOpa1^+/+^* and *dOpa1^+/−^* treated with antioxidant were similar (RI = 0.107, RI = 0.133, p>0.05) (data not shown). In the presence of light, antioxidant treatment made no difference in wild-type *dOpa1^+/+^* (RI = 0.667), while mutant *dOpa1^+/−^* showed a significant increase in phototatic response (RI = 0.427, p<0.05, Figure 3C). These results suggest that antioxidant treatment can partially reverse abnormal visual response in *dOpa1* mutants. These reversals were observed both in heterozygous mutant *dOpa1^+/in3^* and *dOpa1^+/ex2^*. The partial reverse of ERG response and phototaxis of antioxidant treatment support our hypothesis that ROS plays an essential role in the pathogenesis of optic atrophy and antioxidants have the potential to be an effective therapeutic agent for optic atrophy.

### Heterozygous mutation of *dOpa1* results in decreased heart rates and increased cardiac arrhythmias that were not affected by antioxidant treatment

**Figure 4:**
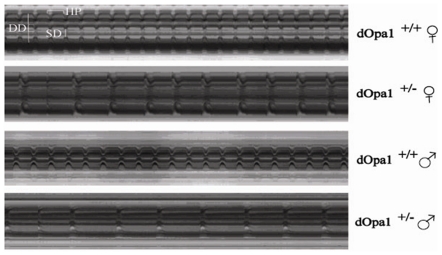
Representative M-mode traces (10 s) of semi-intact *Drosophila* from different genotype and sex. M-modes were created by electronically “cutting” out a single specified vertical row of pixels that span the heart tube from every frame of the movie, and aligning them horizontally. The M-modes describe the vertical movement of the heart walls in time. In these representative M-modes of 3-week-old *dOpa1^+/+^* and *dOpa1^+/−^ Drosophila*, it is seen that mutation causes increased heart period, due primarily to increased diastolic intervals. The dilation of mutant heart tube is also visible.

**Figure 5:**
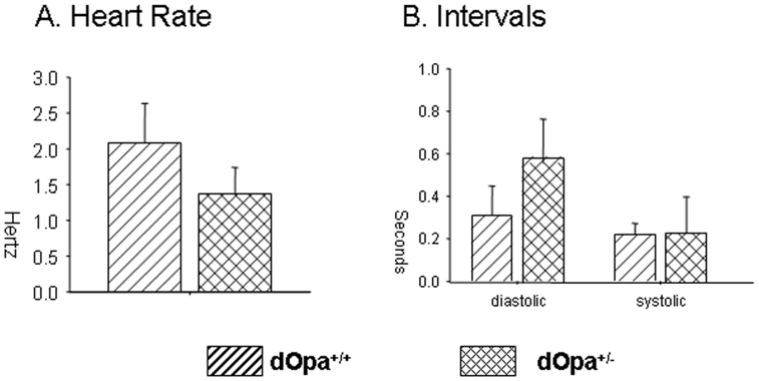
A. Heterozygous mutation of *dOpa1* results in decreased heart rate (p<0.001). Single dashed bars represent *dOpa1^+/+^* and double dashed bars represent *dOpa1^+/−^ Drosophila*. Error bars are standard deviation of the mean. Heart rates were recorded at 3 weeks of age. Figure 5B. The decreased heart rate shown in Figure 5A was predominantly due to an increase in diastolic intervals (<0.001), not an increase in systolic intervals (0.09).

Heterozygous mutation of *OPA1* has predominantly been associated with retinal disorders, but recent studies indicate that it disrupts function of other organs [27], [28], [29]. Using our *Drosophila* model, we tested if heterozygous *dOpa1* mutation affects cardiac function using the methods described by Ocorr et al.[21] Since in intact *Drosophila*, the signals from the central nervous system (CNS) may affect the rate and rhythm of the heart, we removed the *Drosophila* head, ventral thorax, ventral abdominal cuticle and all internal organs as described previously [21]. *Drosophila* hearts were then exposed and video recorded for 30 seconds (Figure 4). The movies were analyzed using the software as described in Ocorr et al.[21]. Forty *Drosophila* of each genotype (20 male and 20 female) were tested at week 3. As shown in Figure 5, heart rate was significantly reduced in heterozygous *dOpa1* mutants (Figure 5, A). The decreased heart rates resulted predominantly from increased diastolic intervals, not from changes in systolic intervals (Figure 5, B). Heterozygous*dOpa1* mutation also caused an increase in heart arrhythmias as shown in Figure 5, C.

There is a sexual dimorphism in overall heart size with female hearts generally being larger than those of males, as has been previously reported[22]. Our results show that heterozygous *dOpa1*mutations cause a significant dilation of the heart tube during diastole (*dOpa1* ^+/+^ DD = 67.4+/−11.8 vs. *dOpa1* ^+/−^ DD = 76.6+/−13.9, p<0.05) and during systole (*dOpa1* ^+/+^ SD = 38.6+/−9.9 vs. *dOpa1^+/−^* SD = 46.7+/−12.3, p<0.05). We calculated the percent fractional shortening (FS) from the heart diameter measurements as an estimate of cardiac contractility (33). These results showed that heterozygous *dOpa1* mutations led to a significant reduction in FS (*dOpa1^+/+^* FS = 43.2%+/−7.8% vs. *dOpa1^+/−^* FS = 39.5%+/−6.7%, p<0.05) suggesting a loss of myocardial contractility. In order to determine if excess ROS production played a role in these observed cardiac abnormalities, we treated the *Drosophila* with antioxidant (100 µM MnTBAP). Our results show that antioxidants had no effect on any of the heart function parameters we measured (Data not shown) in either wild-type or heterozygous *dOpa1*mutants. This suggests that changes in ROS production do not mediate the observed effects of OPA mutations on the heart. Instead, heterozygous mutation of *dOpa1* leads to a respiratory defect in Complex II and III of the electron transport chain (ETC) as shown in our recent publication [23].

### Heterozygous mutation of *dOpa1* results in increased heart failure in response to electrical pacing in *Drosophila*

**Figure 6:**
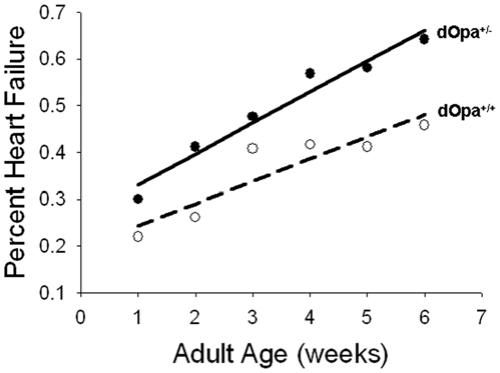
Heterozygous mutation of *dOpa1* results in increased heart failure (solid line) compared to controls (dashed line). Heart failure here is defined as cardiac arrest or fibrillation after a two-minute recovery period following electrical pacing. Heart failure increased with age for both controls and mutants, but was significantly higher in mutants. 40 males and 40 females of each genotype were tested at every age.

We also examined the effect of stress on cardiac function in *Opa 1* mutants. 80 *Drosophila* of each genotype (40 male and 40 females) were tested for heart failure in response to electrical pacing for six consecutive weeks starting at one week of age. Fly hearts were paced with external electrical pacing and heart function was observed immediately following and two minutes after pacing. The effect on heart function was defined as “heart failure” if the heart did not resume a normal beating pattern after a two minute rest period following electrical pacing. Our result showed that heart failure in response to electrical pacing increased with age in all flies tested (Figure 6). However, heterozygous *dOpa1*mutation caused a significant increase in heart failure when compared to wild-type controls and this was true for both males and females (Figure 6, p = 1.63e-05), suggesting heterozygous *dOpa1*mutations compromise heart function in the flies and decrease their ability to tolerate arrhythmia by stress.

### Heterozygous mutation of *dOpa1* results in reduced escape response after stress

**Figure 7:**
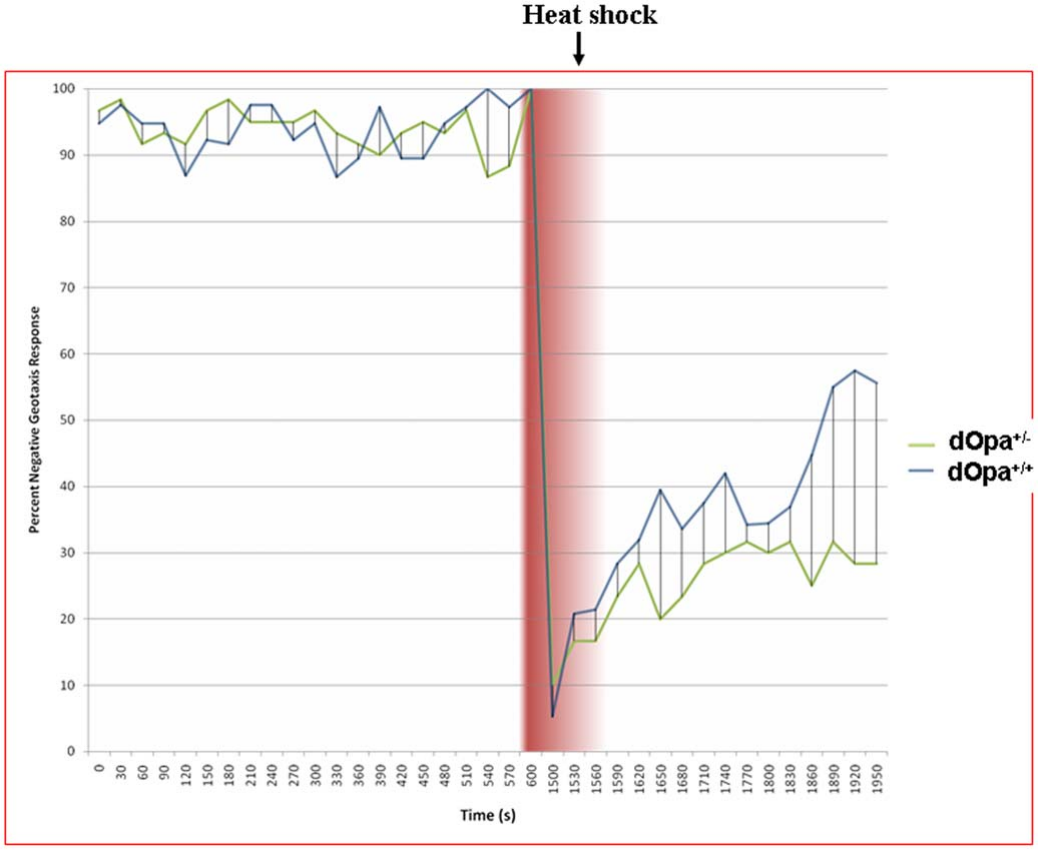
*dOpa1* mutation impairs the ability of *Drosophila* to exhibit a negative geotaxis response following a heat shock. Seven d.o. *Drosophila* were places in 9×2 cm tubes with a line drawn horizontally 1 cm from the base. Every 30 seconds, the tubes were tapped until all *Drosophila* were at the bottom of the tubes. A baseline locomotive index (number of *Drosophila* above the 1 cm line after 15 seconds) was established at 22°C. *Drosophila* were then subject to a 20 minute 37°C heat shock and returned to 22°C. Subsequently, the locomotive indexes were recorded as the samples recovered.

To determine the effect of *dOpa1* mutation on *Drosophila* physical fitness, we quantified the escape responses of *dOpa1^+/−^* and *dOpa1^+/+^* using a previously described technique [24]. Under non-stress conditions, there were no significant differences between *dOpa1^+/−^* and *dOpa1^+/+^* for all age groups (data not shown). We then tested if heat-shock stress had an impact on the escape response of *dOpa1^+/−^* and *dOpa1^+/+^ Drosophila*. For this experiment, *Drosophila* in vials were startled by tapping them to the bottom of the vial. The climbing capacity, before and after a 10-min 37°C heat shock, was determined by calculating the percentage of flies that climbed up>1.0 cm within 15 seconds (locomotive index). The time required for the locomotive index to exceed 50% (recovery time) was also determined (Figure 7). There were no differences in climbing capacity before the heat shock. However, the *dOpa1^+/−^* mutants exhibited lower locomotive indexes after the heat shock and were unable to recover within one hour. In contrast, the *dOpa1^+/+^ Drosophila* recovered within ∼10 minutes (Figure 7). These results suggest that under stress, mutation of *dOpa1* causes decreased physical fitness. For cardiac and skeletal dysfunction, previously two studies showed that OPA1 in humans causes mitochondrial DNA deletion [28], [29]. We have tested this in our *Drosophila* model. As shown in Supplemental Figure S1, we see no difference DNA deletion in heterozygous mutant fly compared with the wild-type. We also used a long range PCR to test the deletion. The results are very similar (Data not shown). Instead, heterozygous mutation of *dOpa1* leads to a respiratory defect in Complex II and III of the electron transport chain (ETC)[23].

## Discussion

Mutation of *OPA1* can lead to DOA and is one of the most common genetic causes of degeneration of retinal ganglion cells, leading to blindness. The main clinical features of DOA include reduced visual acuity, color vision abnormalities, centrocaecal visual field defects and pallor of the optic nerve head. However, the *OPA1* gene is ubiquitously expressed and recent studies have suggested that mutation of OPA1 results in complicated optic atrophy “plus” phenotypes. These clinical phenotypes include optic atrophy, as well as neurosensorial hearing loss, ataxia, sensory motor polyneuropathy and chronic progressive external ophthalmoplegia. In skeletal muscle of optic atrophy patients, mutation of OPA1 causes mitochondrial myopathy characterized by cytochrome c oxidase negative, ragged red fibers and abnormal morphology and distribution of mitochondria [25]. Since both the heart and skeletal muscle are the high energy-demanding organs, mutations of *OPA1* may affect the cardiac and skeletal function, which prompted us to analyze function of multiple organs in our *Drosophila dOpa1* mutant model. Here we show that heterozygous *dOpa1^+/−^* mutations not only perturb eye function, but also decrease cardiac function and decrease escape responses under stress. These results are consistent with recent studies that OPA1 mutation affects other organs besides the retina [18], [19], [20], [21], [22], [23], [24], [27], [28], [29].

An electroretinogram (ERG) is an extracellular recording of the compound eye and their synaptic targets, the laminar neurons [19], [20]. With an orange light stimulus, a typical wildtype ERG consists of a sustained corneal-negative photoreceptor component and transient components at the onset and offset of the light stimulus. Thus, ERGs can be used to detect defects in both the photoreceptor response and the response of laminar neurons. The abnormal on-transient but normal photoreceptor component obtained with the *dOpa1^+/−^ Drosophila* suggest that photorecptor function is normal but either laminar neuron function or synaptic transmission between the photoreceptor and the laminar neuron is impaired. Based on the correlation with age and the fact that antioxidants partially rescued the phenotype of ERG and vision loss, our results suggest that high ROS levels contribute to the phenotypes, which is consistent with our previous results that somatic mutation of dOp1 may cause increased ROS production [18].

A number of studies have implicated mitochondrial defects in cardiac dysfunction. Mitochondrial dysfunction can cause decreased ATP production and/or increased ROS production, and either outcome can lead to cardiac cellular damage. Mutations affecting mitochondrial function have been shown to cause Leber's Hereditary Optic Neuropathy (LHON), MERRF, MELAS, Chronic Progressive External Ophthalmoplegia (CPEO), Kearns-Sayre Syndrome (KSS), and various other pediatric and adult cardiomyopathies. In Friedreich ataxia, mutations occur in the nuclear frataxin gene. Frataxin encodes a mitochondrial inner membrane protein, which transports iron out of the mitochondrial matrix. In the absence of frataxin, iron accumulates in the mitochondria, stimulating the Fenton reaction and ROS production [26], [27]. However, our result show that antioxidants do not improve cardiac abnormalities caused by *dOpa1* mutations, suggesting that these mutations result in cardiac dysfunction primarily by affecting mitochondrial ATP production rather than by increasing ROS production.

Mitochondrial dysfunction has been associated with many skeletal abnormalities such as MERRF. Recently, two groups reported that several OPA1 mutations cause “optic atrophy plus syndrome”. In addition to optic atrophy, the clinical phenotypes of “optic atrophy plus syndrome” was reported [27], [28], [29]. The mitochondrial diseases MERRF, and MELAS are cuased by mutations of the mitochondrial genome, but associated with abnormal proteolytically processing of OPA1 [17]. Taken together, this supports the hypothesis that OPA1 mutations cause skeletal abnormalities [27], [28], [29]. Our result showed that heterozygous *dOpa1* mutation caused reduced escape response, suggesting the possibility of abnormal function of the skeletal muscles.

The analysis of the effect of *dOpa1^+/−^* on *Drosophila* organ systems showed that organ dysfunction developed in an age-dependant manner, consistent with the clinical optic atrophy phenotype. In humans, the onset of most optic atrophies is in childhood and the pathologies typically include progressive bilateral loss of visual acuity, abnormal color vision [5]–[8], and temporal pallor of the optic disk [28]. The loss of visual acuity is caused by a decrease in the number of optic nerve fibers in the central retina. The age-dependent phenotypes suggest cumulative damage and this is consistent with our observation that *dOpa1* mutation results in increased ROS production and accumulation in eyes[18]. Together, these observations partially explain the significant intra- and inter-familial variations in vision loss, the incomplete penetrance of the disease and sex dependent phenotype [9,16,17,41] since estrogen receptors in mitochondria may play an antioxidant contributing to the observed sex influenced eye phenotypes [29].

Dominant optic atrophy is one of the most common forms of inherited optic neuropathy [5]. The etiology is heterogeneous and many inheritance patterns have been described, including autosomal dominant, autosomal recessive, X-linked and mitochondrial. Importantly, several other optic atrophies share pathophysiology with DOA and, therefore, the results from this study can be extended to other types of optic atrophy and neurodegenerative disease. Moreover, mutation of *OPA1* causes mitochondrial dysfunction, including fragmentation, decreased ATP production, increased ROS production and decreased mitochondrial membrane potentials [10]. Abnormalities of mitochondrial fusion and fission have been reported in several mitochondrial disorders [43,44]. Together, this suggests that OPA1 mutations cause multiple organ abnormalities, including auditory neuropathy, peripheral neuropathy (apoptosis and ophthalmoplegia), cardiomyopathy and myopathy, suggesting that OPA1 could be a common target for treatment of mitochondria-related disorders.

## Materials and Methods

### Drosophila stocks

y[d2] w[1118] P{ry[+t7.2] = *ey*-FLP.N}2; P{ry[+t7.2] = neoFRT}42D PBac{WH}CG8479^f02779^ (*dOpa1^+/−^*) and y[d2] w[1118] P{ry[+t7.2] = *ey*-FLP.N}2; P{ry[+t7.2] = neoFRT}42D PBac{WH}CG8479^f03594^ (*dOpa1^+/+^*) *Drosophila* were used in this study. The stocks were established in a previous study [18]. *dOpa1^+/−^* mutants and *dOpa1^+/+^* controls were transferred to fresh food every two to three days while aging. *dOpa1^+/−^* ^(antiox)^ *Drosophila* and *dOpa1^+/+^* ^(antiox)^ *Drosophila* were maintained on 100 µM MnTBAP antioxidant food.

### Electroretinograms (ERG)

ERGs were performed as previously described [31] on 7, 14, 21, 28, 35, and 42 d.o. *Drosophila*. A 300-Watt Halogen lamp (OSRAM) with an unattenuated intensity of 810 µW/cm^2^ at the level of the photoreceptors was used for light stimuli. Kodak neutral filters and a Corning orange (Or) filter were used to achieve the desired light intensity and color for the light stimuli. For each stimulus, the *Drosophila* was first dark-adapted for 3 minutes and then given a 1 second light stimulus. Orange light stimuli were used because they elicit larger on-transient amplitudes than white light stimuli. All recordings were made at 25°C. Signals were sampled at 2 kHz with an analog-to-digital converter (Digidata 1200A), and the data were acquired and analyzed using a computer with Axoscope (Axon Instruments).

### Larval phototaxis assay

The phototaxis assay tested larval visual response using methods according to Lilly and Carlson [32]. Plastic petri plates were sectioned into four quadrants, two dark quadrants diametrically opposite to two clear quadrants. 20 ml of 1% agarose was poured into the clear quadrants, and 20 ml of 1% agarose containing 0.6% charcoal powder was poured into the dark quadrants. After the plates cooled to room temperature, 15 ml of 1% agarose was poured evenly on the gel to create a smooth surface. The plates were allowed to equilibrate to room temperature overnight. Thick black papers were used to cover the sides of the plates, and to cover underneath the plates in regions of the dark quadrants to reduce light reflection. 50 second-instar larvae were placed on the center of the experimental test plate. The plate was placed in total darkness and the larvae were allowed to migrate for 3 minutes for the control. Subsequently, the plate was covered with a piece of black foam to reduce light reflection and then it was placed on a light box in a dark room. The larvae were allowed to migrate for 3 minutes. Response index (RI) was calculated by subtracting the number of larvae on the clear quadrants (C) from the number of larvae on the dark quadrants (D) all divided by the total larvae used, RI = (D – C)/(D+C).

### Cardiac function analysis

*Drosophila* were dissected under an oxygenated saline solution that served as artificial hemolymph, composed of NaCl_2_ (108 mM), KCl (5 mM), CaCl_2_ (2 mM), MgCl_2_ (8 mM), NaH_2_PO_4_ (1 mM), NaHCO_3_ (4 mM), HEPES (15 mM), sucrose (10 mM), trehalose (5 mM), pH 7.1,as described previously [34,47,48] A Hamamatsu EM-CCD digital camera (McBain Instruments,Chatsworth, CA) mounted on a Leica DM LFSA microscope with a 10 x water immersion lens (McBain Instruments, Chatsworth, CA) and Simple PCI image capture software (Compix Imaging System, Selwicky, PA) was used to digitally record beating hearts in semi-intact *Drosophila* preparation [21]. All movies were taken 15–45 minutes following dissection, were 30 seconds long, and were taken as frame rates of approximately 130 frames per second. The movement of the heart was analyzed using a previously described software [34,47]. The software tracks movement of the heart edges using both changes in average light intensity of each frame and changes in the intensity in each individual pixel from frame to frame. M-modes were created by the software by electronically “cutting” out a single specified vertical row of pixels that span the heart tube from every frame of the movie, and aligning them horizontally. The M-modes describe the vertical movement of the heart walls in time. Diastolic intervals were measured as the pause in movements occurring during relaxation. The time between the ends of two consecutive diastolic intervals gave the heart period, and the difference between the heart period and the diastolic intervals gave the systolic interval. The arrhythmicity index (AI) is the heart period standard deviation normalized to the median heart period to compensate for variabilities between flies and for effects on periodicity due to prolonged contractions[21]. Diastolic and systolic diameters were measured by marking the edges of the heart tube in maximum contraction and maximum relaxation states in manually advanced movies. Markings were made in the third abdominal segment, which is a relatively linear region of the heart tube. Percent fractional shortening was calculated as (Diastolic diameter – Systolic diameter)/Diastolic diameter. % FS is the fraction of relaxed diameter size that the heart shortens to when it contracts and thus gives an estimate of the contractibility of the heart tube. We tested for population and sex effects and population by sex interactions by ANOVA in R (www.r-project.org).

### Cardiac pacing assay

*Drosophila* heart function was tested using an electrical pacing assay as previously described[49]. Briefly, *Drosophila* were anesthetizes by exposing to triethylamine (FlyNap™; Caroline Biological, Burlington, NC, USA), and were placed on glass microscope slides with their heads and posterior abdomens touching two strips of conductive electrode gel. A square wave stimulator was used to pace the heart at 40V and 6 Hz for 30 s. The assay was done under an inverted microscope and an X25 objective. Each *Drosophila* was visually checked for cardiac arrest or fibrillation immediately after pacing and then two minutes after pacing. Data were analyzed using a Chi-square test.

### Locomotive Assay

*Drosophila* were placed in 9×2 cm tubes with a line drawn horizontally 1 cm from the base. Every 30 seconds, the tubes were tapped until all *Drosophila* fell to the bottom of the tubes. A baseline locomotive index (number of *Drosophila* above the 1 cm line after 15 seconds) was established at 22°C. The *Drosophila* were then subjected to a 20 minute 37°C heat treatment and returned to 22°C. The locomotive indexes were recorded as the samples were allowed to recover [24].

## Supporting Information

Figure S1 dOpa1+/− Drosophila is not associated with major mitochondrial DNA deletions. Total genomic DNA from 3 wk and 6 wk old dOpa1+/− and dOpa1+/+ flies were fractionated by agarose gel electrophoresis,visualized by ethidium bromide staining and then transferred to nylon membrane. Digoxigenin-tagged DNA probe (Long PCR products spanning positions 1,867–14,744 of the Drosophila mtDNA (Panel A) were generated by random primer labeling, hybridized to target sequences, bound by anti-digoxigenin-AP, Fab fragments, and then detected with CDP-Star (Roche). Panel B, Southern blot for mitochondrial genome, showing a very similar pattern in dOpa1+/− and dOpa1+/+ (Panel B).
(0.40 MB DOC)
