# Supplementary figures and images for: Heterozygous Mutation of Drosophila Opa1 Causes the Development of Multiple Organ Abnormalities in an Age-Dependent and Organ-Specific Manner
Source: PLoS One. 2009 Aug 31;4(8):e6867. doi: 10.1371/journal.pone.0006867 (PMC2730818; doi:10.1371/journal.pone.0006867)

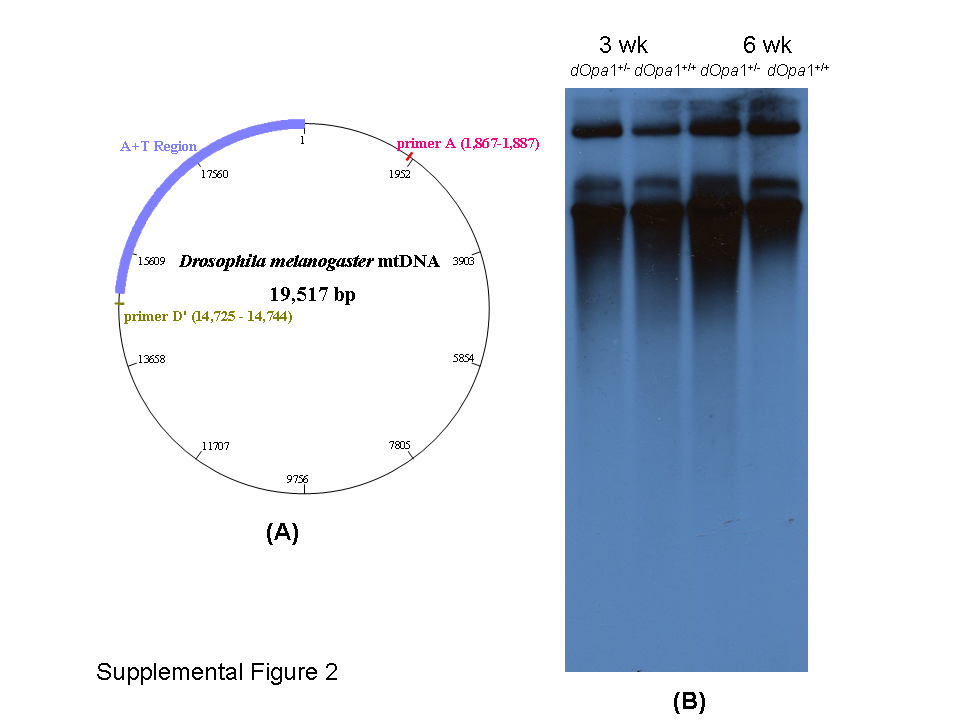

Supplement: Figure S1 — dOpa1+/− Drosophila is not associated with major mitochondrial DNA deletions. Total genomic DNA from 3 wk and 6 wk old dOpa1+/− and dOpa1+/+ flies were fractionated by agarose gel electrophoresis,visualized by ethidium bromide staining and then transferred to nylon membrane. Digoxigenin-tagged DNA probe (Long PCR products spanning positions 1,867–14,744 of the Drosophila mtDNA (Panel A) were generated by random primer labeling, hybridized to target sequences, bound by anti-digoxigenin-AP, Fab fragments, and then detected with CDP-Star (Roche). Panel B, Southern blot for mitochondrial genome, showing a very similar pattern in dOpa1+/− and dOpa1+/+ (Panel B). (0.40 MB DOC) [file pone.0006867.s001.tif]
